# 2D Ti_3_C_2_T_x_ MXene nanozymes based electrochemical MicroRNA biosensor and application to early diagnosis of sepsis-associated acute kidney injury

**DOI:** 10.1007/s00604-025-07559-2

**Published:** 2025-11-01

**Authors:** Xiangyu Deng, Xia Zheng, Anyi Chen, Yuqing Li, Siling Chen, Jiangling Wu, Jianjiang Xue, Rongjun Yu, Min Zhao, Jingfu Qiu

**Affiliations:** 1 Department of Clinical Laboratory, University-Town Hospital of Chongqing Medical University, Chongqing, 401331 China; 2 College of Public Health, Chongqing Medical University, Chongqing, 400016 China; 3 Key Laboratory of Clinical Laboratory Diagnostics (Ministry of Education), College of Laboratory Medicine, Chongqing Medical University, Chongqing, 400016 China

**Keywords:** Ti_3_C_2_T_x_ MXene, Nanozymes, MicroRNA, Electro-catalysis, Cascading catalytic amplification, Electrochemical biosensor

## Abstract

MicroRNA detection-based molecular diagnostic methods offer a favorable pathway for the early diagnosis of sepsis-associated acute kidney injury (SA-AKI), which is crucial for reducing mortality and improving patient outcomes. In this work, a highly sensitive electrochemical biosensor was fabricated for the determination of serum miR-452-5p, an emerging biomarker for SA-AKI, leveraging the novel Ti_3_C_2_T_x_ MXene nanozymes and catalytic hairpin assembly isothermal amplification. Surprisingly, the two-dimensional Ti_3_C_2_T_x_ MXene nanozymes demonstrated remarkable electro-catalytic activity towards choline, wihch was attributed to the strong adsorption interaction between the oxygen atoms on the Ti_3_C_2_T_x_ MXene surface and the hydroxyl group of choline. Based on above-mentioned characteristic of Ti_3_C_2_T_x_ MXene nanozymes, a cascading catalytic amplification strategy by integrating Ti_3_C_2_T_x_ MXene with alkaline phosphatase using phosphorylcholine chloride as the substrate was designed, thereby achieving highly efficient electrochemical signal amplification. The electrochemical biosensing approach exhibited remarkable sensitivity for miR-452-5p detection, with a linear response over a concentration range from 0.1 fM to 10 nM and a low detection limit of 0.09 fM. Additionally, the developed electrochemical biosensor for direct serum miR-452-5p detection present a diagnostic efficiency of 96% in clinical samples analysis, highlighting its strong potential for the early detection and risk assessment of SA-AKI.

**Graphical Abstract:**

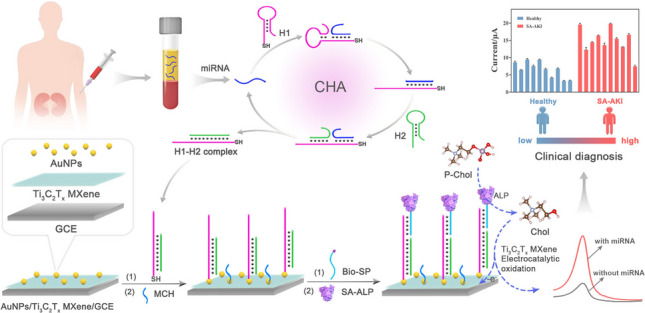

**Supplementary Information:**

The online version contains supplementary material available at 10.1007/s00604-025-07559-2.

## Introduction

Approximately 50% of acute kidney injuries originate from sepsis, correlated with alarmingly elevated mortality rates attributed to deficiencies in early diagnosis and intervention [1, 2]. Current diagnosis of sepsis-associated acute kidney injury (SA-AKI) is mainly achieved through the detection of urine volume and serum creatinine, which severely lag behind the disease progression and lead to poor outcomes [3, 4]. As endogenous small non-coding RNA molecules, microRNAs (miRNAs) perform essential gene regulatory functions within cellular environments by modulating protein-coding genes [5–7]. Recent evidences has showed that miRNAs are promising biomarkers for indicating SA-AKI, as significantly differential expression of miRNAs has been observed in the blood of patients with SA-AKI [8–10]. Up to date, reverse transcription PCR (RT-PCR) is the most widely used method for miRNAs detection in clinical laboratory [11]. However, due to the short chain length and highly similar sequence within a family, RT-PCR lacks sufficient accuracy for low-abundance miRNAs detection, thereby limiting its broader application in clinical diagnosis [12]. Thus, there is an urgent need for an accurate and sensitive miRNA detection method to fulfill the current requirements of the early clinical diagnosis of SA-AKI.

The electrochemical biosensor has emerged as a prominent analytical tool in clinical diagnosis due to its exceptional attributes, including high sensitivity, selectivity and robust quantitative capabilities [13–15]. However, the application of electrochemical biosensors in the detection of low-abundance miRNAs necessitates highly efficient signal amplification strategies [16–18], such as nucleic acid amplification, nanomaterial-catalyzed signal amplification. Nanozymes, a class of nanomaterials exhibiting enzymatic catalytic activities, have become a potential alternative to overcome critical limitations of natural enzymes in bioanalysis [19, 20]. Among the various nanozymes, Ti_3_C_2_T_x_ MXene has garnered widespread attention in the field of food safety [21–23], clinical diagnosis [24–26] and chemotherapy [27, 28]. The unique surface structure of MXene encompasses Ti-F, Ti-OH and Ti=O bonds that provide effective active sites for diverse adsorbates [29], and endow it with enzyme-like catalytic activity. Recent studies have shown that the peroxidase-like and oxidase-like activities of MXene can be attributed to the electron transfer between its surface functional groups and the substrate [30]. Simultaneously, the metallic conductivity and multi-valent state characteristics of MXene enable it to exhibit efficient performance in carrier transfer and redox reactions [31, 32]. Furthermore, our previous research has demonstrated that the electrocatalytic performance of Ti_3_C_2_T_x_ MXene for 1-naphthol oxidation is strictly proportional to the coverage area of the MXene flakes. Inspired by the above-mentioned MXene properties, its large specific surface area, high electrical conductivity, and rich surface chemical groups endow it with significant potential for application in the development of sensitive electrochemical biosensing.

**Scheme 1.**
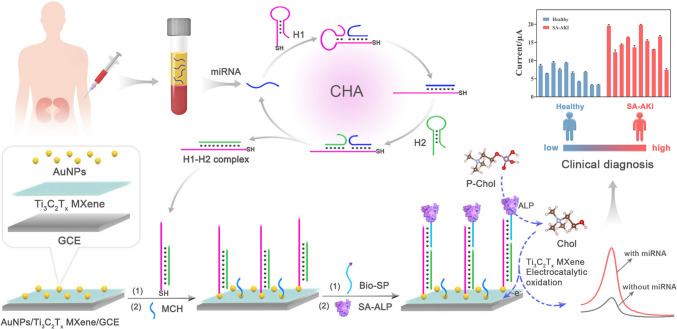
Schematic representation of the electrochemical biosensor for serum miR-452-5p detection based on Ti_3_C_2_T_x_ MXene-mediated cascading catalytic amplification and CHA-mediated isothermal amplification amplification

In this work, we investigated the electrocatalytic activity of Ti_3_C_2_T_x_ MXene toward choline (Chol) oxidation and further employed it to establish a cascading catalytic amplification strategy in combination with alkaline phosphatase (ALP). Notably, Ti_3_C_2_T_x_ MXene exhibited efficient and area-dependent adsorption of Chol on its two-dimensional plane, thereby enhancing the electrochemical oxidation of Chol. On the basis of Ti_3_C_2_T_x_ MXene-mediated cascading catalytic amplification and catalytic hairpin assembly (CHA), a highly sensitive electrochemical biosensor has been developed for precise detection of serum miR-452-5p, a potential biomarker for SA-AKI. As illustrated in Scheme 1, the AuNPs/Ti_3_C_2_T_x_ MXene modified electrode was employed to anchor the hairpin capture probe (H1) *via* a Au-S bond. In the presence of target miR-452-5p, a typical CHA process could be triggered to enable the cyclic assembly of the hairpin probe (H2) onto H1, thereby exposing a toehold to capture the biotin-labeled signal probe (Bio-SP). The fabrication of Bio-SP facilitates the attachment of streptavidin-linked ALP (SA-ALP) to the biosensing interface *via* the typical biotin-streptavidin interaction. Upon the introduction of phosphorylcholine (P-Chol), Chol could be generated through the ALP-catalyzed hydrolysis of P-Chol. Subsequently, the generated Chol undergo electrochemical oxidation on the electrode surface, which is significantly enhanced by the electro-catalytic effect of Ti_3_C_2_T_x_ MXene, thereby improving the electrochemical response. Benefiting from the synergistic effect of CHA-mediated isothermal signal amplification and multi-enzyme cascade amplification, this electrochemical biosensor demonstrated exceptional sensitivity for miR-452-5p detection with a Linear response ranging from 0.1 fM to 10 nM and a limit of detection (LOD) as low as 0.09 fM. This advancement not only provides a robust bioanalytical tool for clinical diagnosis of SA-AKI, but also shows its broad application potential in serum miRNA detection, especially in the early diagnosis of diseases.

## Experimental section

### Reagents and materials

Reagents and materials are listed in the Supplementary Information.

### Peroxidase-like activity assay of Ti_3_C_2_T_x_ MXene

The peroxidase-like activity of Ti_3_C_2_T_x_ MXene was evaluated by quantifying the TMB oxidation reaction catalyzed by H_2_O_2_/Ti_3_C_2_T_x_ MXene system in 0.2 M acetate buffer. After 20-min incubation in a temperature-controlled water bath, the resultant chromogenic TMB product was analyzed *via* UV-Vis spectroscopy, with the characteristic absorbance peak observed at 652 nm (A652). To establish the optimal reaction conditions, Ti_3_C_2_T_x_ Mxene was dispersed in 0.2 M acetate buffer at a concentration of 33.0 μg/mL. Subsequently, 1.3 mL of Ti_3_C_2_T_x_ Mxene suspension was introduced into a mixture containing 100 μL of 5.0 mM TMB substrate and 100 μL H_2_O_2_ (30%, v/v). The steady-state kinetics of the Ti_3_C_2_T_x_ MXene system was conducted. Following addition of TMB and H_2_O_2_ to 33.0 μg/mL MXene suspensions, reactions proceeded for 20 min, with A652 being recorded every 0.2 s.

### Gel electrophoresis characterization of CHA

Native polyacrylamide gel electrophoresis (PAGE) was used to characterize CHA (Fig. S5). In brief, 10 μL of the sample was combined with the loading buffer and subsequently injected into the sample well of 20% gel. Electrophoresis was running on the gel at 80 V for 100 min. After submerging the gel in the chromogenic solution for 30 min, the gel image was captured using a G:BOX F3 documentation apparatus.

### Fabrication of the biosensor and electrochemical measurement procedure

0.3 μm and 0.05 μm alumina slurries were sequentially used to polish the bare glassy carbon electrode (GCE). The electrode was then ultrasonically cleaned with deionized water, anhydrous ethanol, and deionized water again and finally dried under ambient conditions using nitrogen gas. Then, 10 μL of Ti_3_C_2_T_x_ MXene suspension (0.1 mg/mL) was carefully dropped onto the surface of cleaned GCE and allowed to naturally dry at room temperature. Subsequently, 10 μL of AuNPs solution was dropped on the pre-modified electrode (Ti_3_C_2_T_x_ MXene/GCE) and allowed to dry naturally at room temperature, resulting in the formation of the AuNPs/Ti_3_C_2_T_x_ MXene/GCE.

At the same time, 10 µL of thiol modified H1 (10 μM) and 10 µL of H2 (10 µM) were separately heating at 95 °C for 5 min to maintain them in a single-stranded conformation through thermal denaturation, thereby facilitating the subsequent formation of hairpin structures. Later, the solution consisting of 10 μL H1 (10 μM), 10 μL H2 (10 μM) and varying concentrations of target miR-452-5p were incubated at 37 °C for 2 h. After that, the above supernatant solution (10 μL) was dropped onto the surface of AuNPs/Ti_3_C_2_T_x_ MXene/GCE and left to incubate overnight at 4 °C. Then, 10 μL of MCH (1.0 mM) was incubated on the modified electrode for 1 h to block the nonspecific binding sites. Next, 10 µL of Bio-SP (2.0 µM) was incubated on the electrode surface for 1 h. Following buffer rinsing, 10 µL of DEA buffer containing 8 mg/mL BSA and 1.25 µg/mL SA-ALP was incubated on the modified electrode for 30 min. Ultimately, the electrochemical signal was quantified using DPV in the DEA buffer supplemented with 1.0 mg/mL P-Chol.

## Results and discussion

### Characterization of Ti_3_C_2_T_x_ MXene

**Fig. 1:**
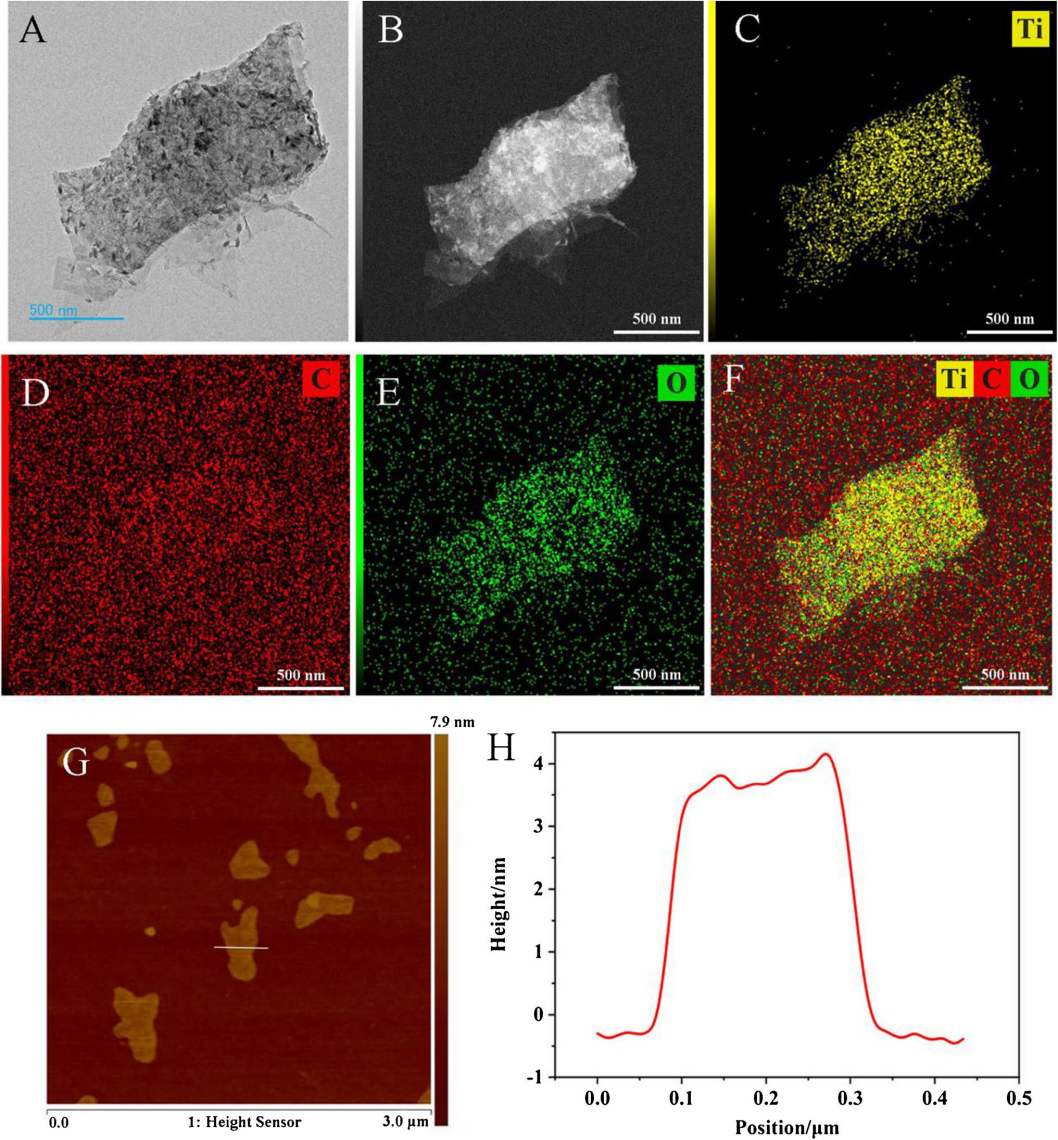
(**A**) TEM image of Ti_3_C_2_T_x_ MXene nanosheet. (**B**) HAADF-STEM image and (**C**-**F**) corresponding EDS mappings of Ti_3_C_2_T_x_ MXene nanosheet. (**G**) AFM image of Ti_3_C_2_T_x_ MXene nanosheet and (**H**) the corresponding height profile along the white line in AFM image

The morphological characteristic of the Ti_3_C_2_T_x_ MXene was evaluated using transmission electron microscopy (TEM). Fig. 1A clearly showed that Ti_3_C_2_T_x_ MXene displayed a distinctive sheet-like morphology, with lateral dimensions extending up to 1 µm. The elemental spatial organization within Ti_3_C_2_T_x_ MXene was also resolved through HAADF-STEM coupled with elemental mapping (Fig. 1B-1F). Ti, C and O exhibited well-defined outlines that were in excellent agreement with the HAADF-SEM image (Fig. 1C-1F). Moreover, the elemental composition of the Ti_3_C_2_T_x_ MXene, including Ti, C, and O, was analyzed by energy dispersive X-ray spectroscopy (EDS) (Fig. S1), confirming its consistency with our expectations.

In addition, the morphology and thickness of Ti_3_C_2_T_x_ MXene were investigated using atomic force microscope (AFM). As depicted in Fig. 1G-1H, Ti_3_C_2_T_x_ MXene exhibited a typical layered morphology, with a nanosheet thickness of approximately 4 nm, corresponding to about three layers, which was in agreement with previous literature [33].

### Evaluation of peroxidase-like kinetics of Ti_3_C_2_T_x_ MXene nanozymes

**Fig. 2:**
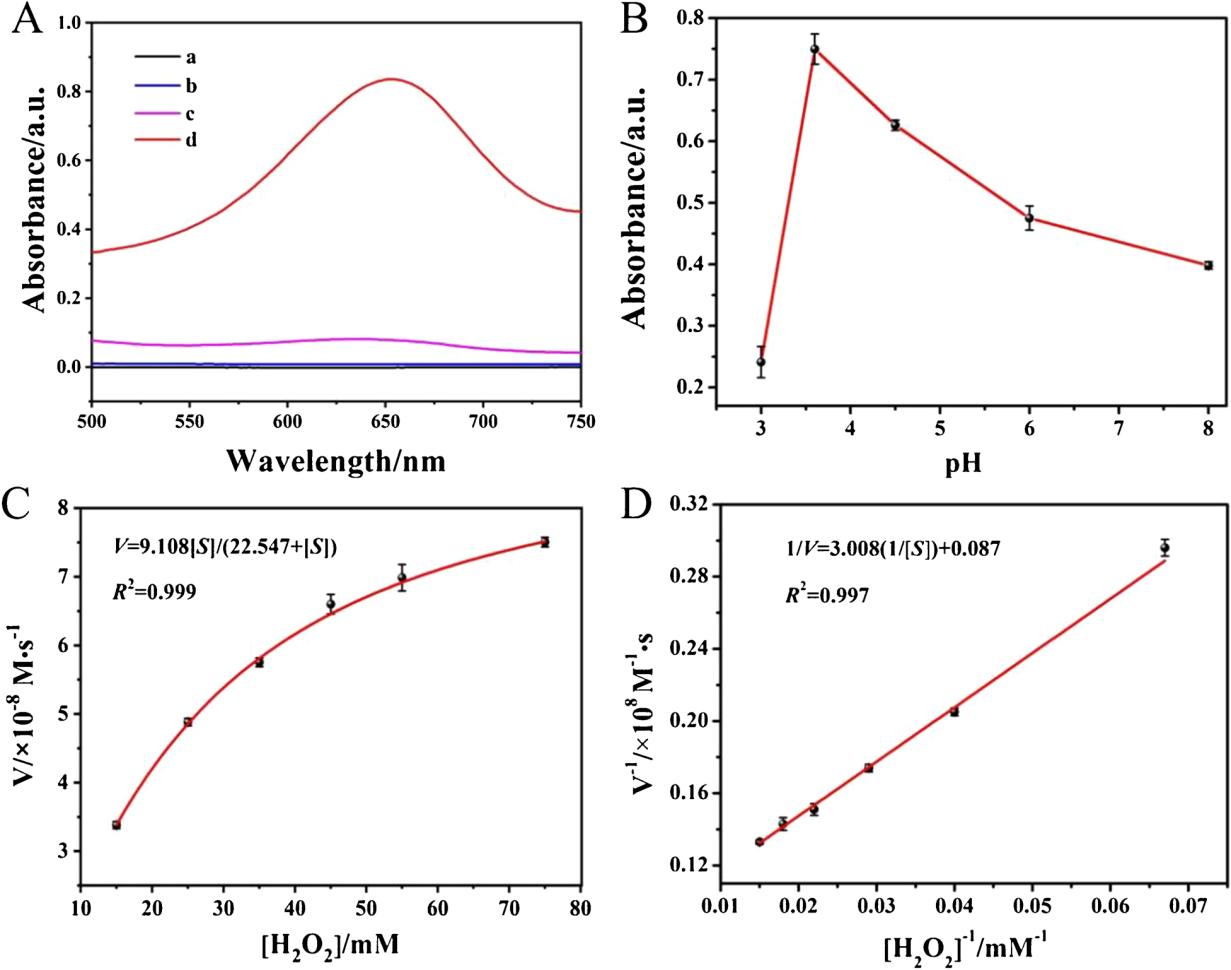
(**a**) UV–Vis absorption spectra of TMB + H_2_O_2_ (a), Ti_3_C_2_T_x_ MXene + TMB (**b**), Ti_3_C_2_T_x_ MXene + H_2_O_2_ (**c**), and Ti_3_C_2_T_x_ MXene + TMB + H_2_O_2_ (**d**) reaction systems. (**B**) UV–Vis absorption spectra of varied pH conditions. (**C**) Steady-state kinetics assay and (**D**) corresponding double-reciprocal plots of Ti_3_C_2_T_x_ MXene (33.0 µg/mL) with different concentrations of H_2_O_2_ (15, 25, 35, 45, 55, 75 mM) with fixed TMB concentration (0.33 mM)

The peroxidase-like activity of Ti_3_C_2_T_x_ MXene was evaluated based on typical chromogenic TMB-H_2_O_2_ system. Upon oxidation, the solution exhibited a bluish hue attributed to the oxidized tetramethylbenzidine product (oxTMB), which displayed a characteristic UV–Vis spectral signature with maximum absorption at 652 nm, corresponding to its distinct coloration [34]. As presented in Fig. 2A, the incorporation of Ti_3_C_2_T_x_ MXene into the system led to the appearance of a characteristic absorption maximum at 652 nm in UV–Vis spectrum, while no color change or characteristic absorption peaks were observed in other control experiments, including H_2_O_2_ + TMB (curve a), Ti_3_C_2_T_x_ MXene + TMB (curve b), Ti_3_C_2_T_x_ MXene + H_2_O_2_ (curve c). This finding demonstrated that the Ti_3_C_2_T_x_ MXene possessed peroxidase-like catalytic activity, effectively inducing TMB oxidation in the presence of H₂O₂.

In order to optimize peroxidase-like catalytic activity of Ti_3_C_2_T_x_ MXene, pH and temperature were optimized, respectively. As illustrated in Fig. 2B, the most prominent absorbance peak of Ti_3_C_2_T_x_ MXene-TMB-H_2_O_2_ ternary system was observed at pH 3.6. Previous study has indicated that transition metals and their oxides predominantly facilitate H_2_O_2_ decomposition through alkaline pathways under the acidic condition [35]. In theory, peroxidase enzyme (or its nanozyme) selectively cleaved the O-O bond in adsorbed H_2_O_2_ under the acidic condition, generating hydroxyl radicals (•OH) that were adsorbed on the Ti_3_C_2_T_x_ MXene surface. This process was mechanistically attributed to the reduced activation energy barrier for H_2_O_2_ decomposition under the acidic condition. In addition, as shown in Fig. S3, based on the observed maximum absorbance at A652, the optimal catalytic activity of Ti_3_C_2_T_x_ MXene occurred at 37 °C.

Enzymatic kinetics analysis was conducted using the Michaelis-Menten model (Eq. (1)) with rigorous application, further enhanced by the inverse plot conversion as described in the Lineweaver-Burk plot methodology (Eq. (2)) under optimal reaction conditions.

1 $${\text{V}}_{0}\text{=}{\text{V}}_{\text{max}}\left[{\text{S}}\right]/\left({\text{K}}_{\text{m}}\text{+}\left[{\text{S}}\right]\right)$$

2 $${1}/{\text{V}}_{0}\text{=}{\text{K}}_{\text{m}}/{\text{V}}_{\text{max}}\left[{\text{S}}\right]\text{+}{1}/{\text{V}}_{\text{max}}$$

The kinetic parameters were defined as follows: ${\text{V}}_{0}$ denotes the initial reaction velocity; *K*_*m*_ (Michaelis–Menten constant) quantifies the nanozyme-substrate binding affinity, where elevated *K*_*m*_ values inversely correlate with binding strength, ${\text{V}}_{\text{max}}$ represents the maximal enzymatic velocity attainable at saturating substrate concentrations; and $\left[{\text{S}}\right]$ corresponds to the instantaneous substrate concentration.

Specifically, varing concentrations of H_2_O_2_ (15, 25, 35, 45, 55, 75 mM) were added into Ti_3_C_2_T_x_ MXene suspensions (33.0 µg/mL) containing 0.33 mM TMB, to evaluate the H_2_O_2_ concentration-dependent absorption via UV–Vis spectroscopy. As displayed in Fig. 2C-2D, the Michaelis–Menten curves and Lineweaver–Burk plot were obtained, which demonstrated that TMB-H_2_O_2_ concentration was hyperbolically related to the reaction rate. The values of ${\text{K}}_{\text{m}}$ and ${\text{V}}_{\text{max}}$ of Ti_3_C_2_T_x_ MXene for H_2_O_2_ were determined to be 22.547 mM and 9.108 × 10^–8^ M/s, respectively. Similarly, as displayed in Fig. S4, the concentration-dependent absorption of TMB was evaluated using UV–Vis spectroscopy at various concentrations (0.1, 0.3, 0.5, 0.8, 1.0, 1.5, 2.2, 4.0 mM), following the aforementioned method. The values of ${\text{K}}_{\text{m}}$ and ${\text{V}}_{\text{max}}$ of Ti_3_C_2_T_x_ MXene for TMB were determined to be 0.231 mM and 4.944 × 10^–8^ M/s, respectively. Compared with HRP and different kinds of nanozymes (Table S2), Ti_3_C_2_T_x_ MXene exhibited excellent peroxidase-like catalytic activity.

### Characterization of electrode fabrication process

**Fig. 3:**
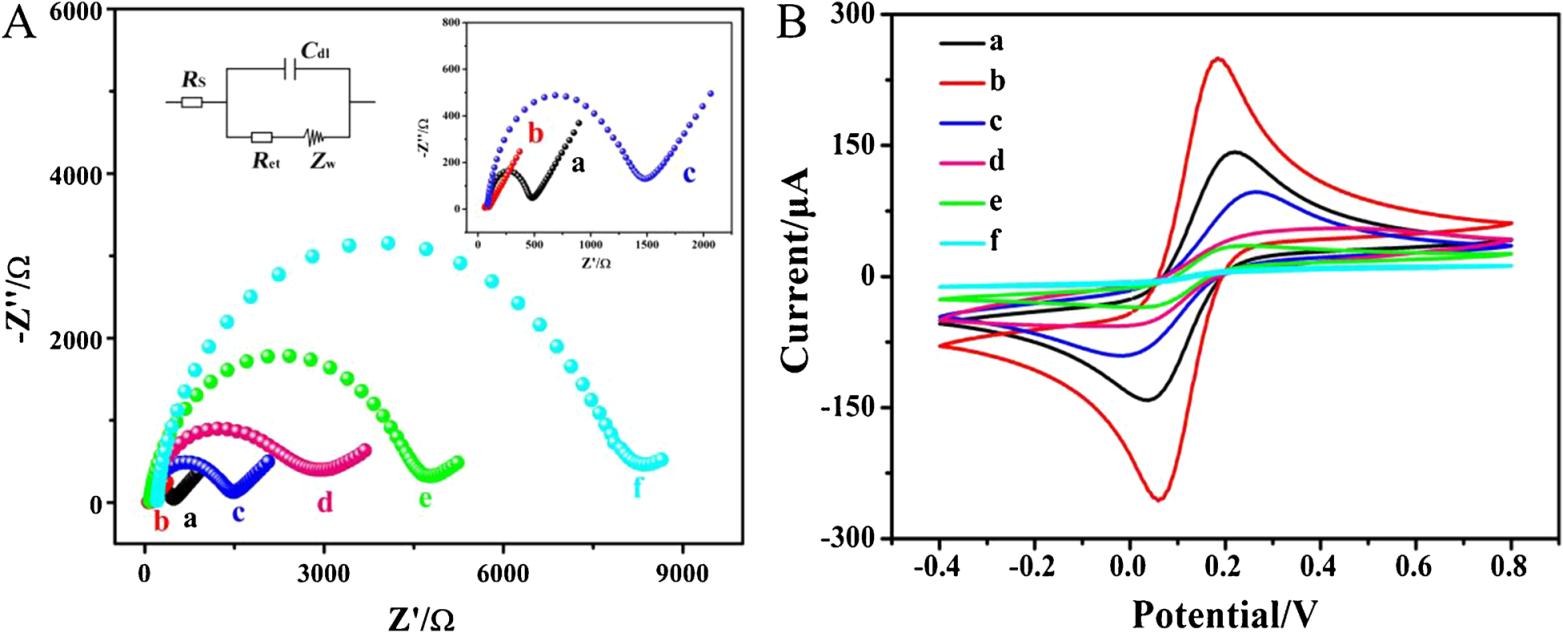
(A) Nyquist plots and (B) CV curves of the different modified electrodes: bare GCE (a), AuNPs/Ti_3_C_2_T_x_ MXene/GCE (b), H1-H2 complex/AuNPs/Ti_3_C_2_T_x_ MXene/GCE (c), MCH/H1-H2 complex/AuNPs/Ti_3_C_2_T_x_ MXene/GCE (d), Bio-SP/MCH/H1-H2 complex/AuNPs/Ti_3_C_2_T_x_ MXene/GCE (e), and SA-ALP/Bio-SP/MCH/H1-H2 complex/AuNPs/Ti_3_C_2_T_x_ MXene/GCE (f). Insert: Amplified Nyquist plots of bare GCE (a), AuNPs/Ti_3_C_2_T_x_ MXene-modified GCE (b) and H1-H2 complex modified GCE (c)

Electrochemical impedance spectroscopy (EIS) and cyclic voltammetry (CV) were employed to monitor the sequential fabrication stages of the biosensor, providing stepwise characterization of the electrode assembly process. As shown in the Nyquist plots of Fig. 3A, the bare GCE demonstrated a diminished semicircular profile in the high-frequency region (curve a), where the radius of the semicircle indicated charge transfer resistance. Upon modification of the bare GCE with AuNPs/Ti_3_C_2_T_x_ MXene, the charge transfer resistance significantly decreased (curve b), indicating that the superior charge transport properties of the AuNPs/Ti_3_C_2_T_x_ MXene to enhance interfacial electron transfer kinetics. Subsequently, a significant charge transfer resistance increase was obtained after the modified electrode was assembled with target-mediated CHA product, dsDNA H1-H2 complex (curve c), which was attributed to the negative charge of nucleic acid backbone that hindered electron transfer between the electrode and [Fe(CN)_6_]^3-/4-^. Next, following the immobilization of MCH on the H1-H2 complex/AuNPs/Ti_3_C_2_T_x_ MXene/GCE, the charge transfer resistance was further increased (curve d), which may be attributed to the ability of MCH to hinder electron transfer to the electrode surface. After the assembly of Bio-SP, a noticeably charge transfer resistance increased was observed (curve e), because the electrostatic adsorption of negatively charged DNA backbones onto the electrode interface. This adsorption process introduced considerable steric hindrance, thereby impeding charge-carrier mobility. Ultimately, biofunctionalization with SA-ALP increased the charge transfer resistance to its maximum value (curve f). Moreover, CV characterization results were consistent with EIS measurements, indicating that both the charge transfer resistance and the current varied systematically with electrode modification (Fig. 3B), thereby confirming the successful and stepwise fabrication of the biosensor, as expected.

### Optimization of experimental conditions

To achieve maximum detection sensitivity and specificity in the biosensing platform, several key experimental parameters were optimized, including the concentration of the Ti_3_C_2_T_x_ MXene, the concentration of H1, the pH of DEA buffer and CHA reaction time. As shown in Fig. S6, the optimal conditions were determined to be 0.1 mg/mL for the Ti_3_C_2_T_x_ MXene concentration, 2.0 µM for H1, the DEA buffer pH of 9.6, and the CHA reaction time of 2 h, respectively.

### Analytical performance of the developed electrochemical biosensor

**Fig. 4:**
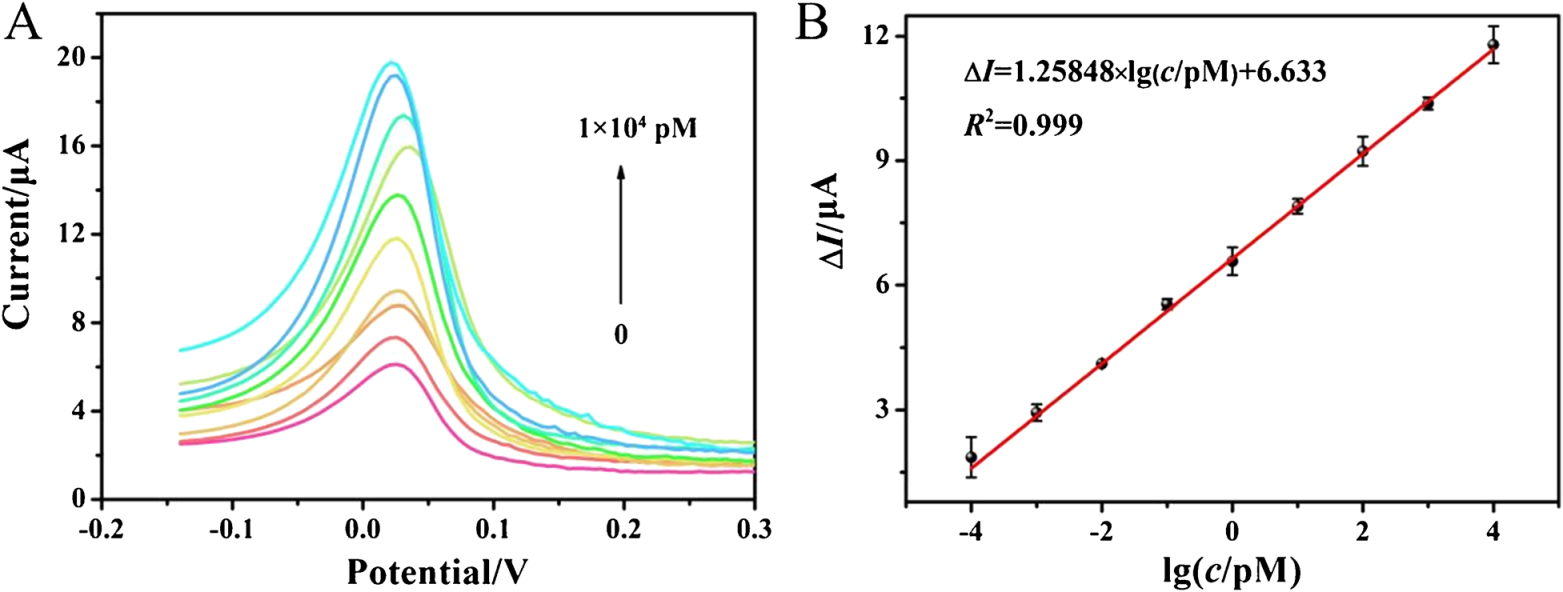
Evaluation of the sensitivity of the developed electrochemical biosensor for miR-452-5p detection. (**A**) DPV curves of the biosensor with different concentrations of miR-452-5p (0 fM, 0.1 fM, 1.0 fM, 10 fM, 100 fM, 1 pM, 10 pM, 100 pM, 1 nM, and 10 nM). (**B**) Calibration curve for delta current *vs.* the logarithm of miR-452-5p concentration, ranging from 0.1 fM to 10 nM

Under the optimal conditions, the DPV signal response was measured at different concentrations of miR-452-5p to evaluate the analytical performance of the biosensor. Fig. 4A showed that the DPV current increased with increasing miR-452-5p concentration ranging from 0.1 fM to 10 nM. Electrochemical calibration curves demonstrated a robust linear relationship between current changes (Δ*I* = *I* - *I*_0_) and the logarithm of miR-452-5p concentrations ranging from 0.1 fM to 10 nM, yielding a pearson correlation coefficient of 0.999. The linear regression equation was expressed as ∆*I*=1.25848×lg(*c*/pM)+6.633, where *c* and ∆*I* represented the miR-452-5p concentration and its corresponding DPV current change (Δ*I* = *I* - *I*_0_), respectively. Here, *I*_0_ and *I* represent the peak current of the DPV signal before and after the introduction of the target miR-452-5p. The LOD was was calculated to be 0.09 fM. Additionally, a comparison with other miRNA detection methods was presented in Table S3, highlighting the superior sensitivity of this proposed biosensing method for miRNA detection, which could be attributed to the excellent catalytic performance of Ti_3_C_2_T_x_ MXene nanozymes.

### Specificity, reproducibility and stability of the developed biosensor

**Fig. 5:**
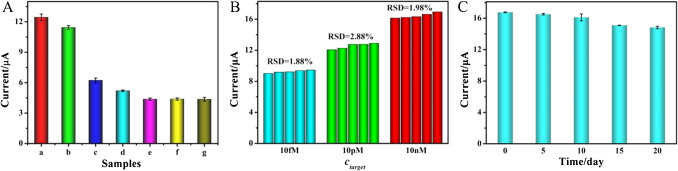
(**A**) Specificity of the biosensor towards miR-452-5p (**a**), mixture (**b**), miR-21(c), miR-146 (d), M1 (**e**), M2 (**f**) and blank (**g**). The concentrations of interference substance and target miR-452-5p were 50 pM and 10 pM, respectively. (B) Reproducibility of the designed biosensor with different concentrations of miR-452-5p (10 fM, 10 pM and 10 nM). (C) Stability of the designed biosensor with different storage days (5, 10, 15, and 20 days). Error bars appearing throughout the manuscript represent standard deviations of three repeated experiments

Furthermore, various miRNAs, including 50 pM single-base mismatched miRNA (M1), 50 pM double-base mismatched miRNA (M2), 50 pM miR-146 and 50 pM miR-21, were introduced as potential interference substances to further evaluate the specificity of the biosensor for target miR-452-5p (10 pM). As shown in Fig. 5A, the proposed biosensor possessed a comparable high current response to the target miR-452-5p (10 pM) and a mixture containing 10 pM miR-452-5p, 50 pM miR-146 and 50 pM miR-21. In contrast, no significant difference was observed for only M1, M2, miR-21, or miR-146 when compared to the blank group, thereby confirming the high specificity of the developed biosensor.

The manufacturing reproducibility of the designed biosensor was identified by measuring the current response to miR-452-5p at three distinct concentrations: 10 fM, 10 pM, and 10 nM. As demonstrated in Fig. 5B, after five independent measurements, the electrodes displayed comparable current outputs. The relative standard deviation (RSD) values of the detection of miR-452-5p were 1.88%, 2.88%, and 1.98%, respectively, suggesting that the designed biosensor had excellent reproducibility.

In order to evaluate the stability of the proposed biosensor, the modified electrodes were stored at 4 °C prior to use. As illustrated in Fig. 5C, no significant differences were observed during the initial 5 days of storage, with current variations remaining below 1.44%. After a storage period of 20 days, the fabricated biosensor preserved 88.44% of its original current signal, demonstrating that the developed biosensor exhibited adequate stability.

### Application of the developed biosensor in actual samples

The clinical applicability of the developed biosensor was assessed in complex biological matrices by spiking diluted serum specimens (10-fold) with varying miR-452-5p concentrations, followed by detection using the developed biosensing method. The detection results of miR-452-5p in human serum samples were summarized in Table 1. Satisfactory recovery values ranging from 95.06% to 103.28% were obtained, with RSDs ranging from 0.21% and 3.22%. These results demonstrated that the proposed electrochemical biosensing method exhibited excellent sensitivity and accuracy in the analysis of actual samples.

**Table 1:** Recovery results of miR-452-5p in serum samples obtained by the developed electrochemical biosensing method (n = 3)

Samples	Added (pM)	Found (pM)	Recovery (%)	RSD (%)
1	0.005	0.0049	97.00	3.16
2	0.050	0.0513	102.60	0.21
3	0.500	0.507	101.40	1.21
4	5	4.753	95.06	2.86
5	50	51.640	103.28	1.30
6	500	504.661	100.93	3.22

**Fig. 6:**
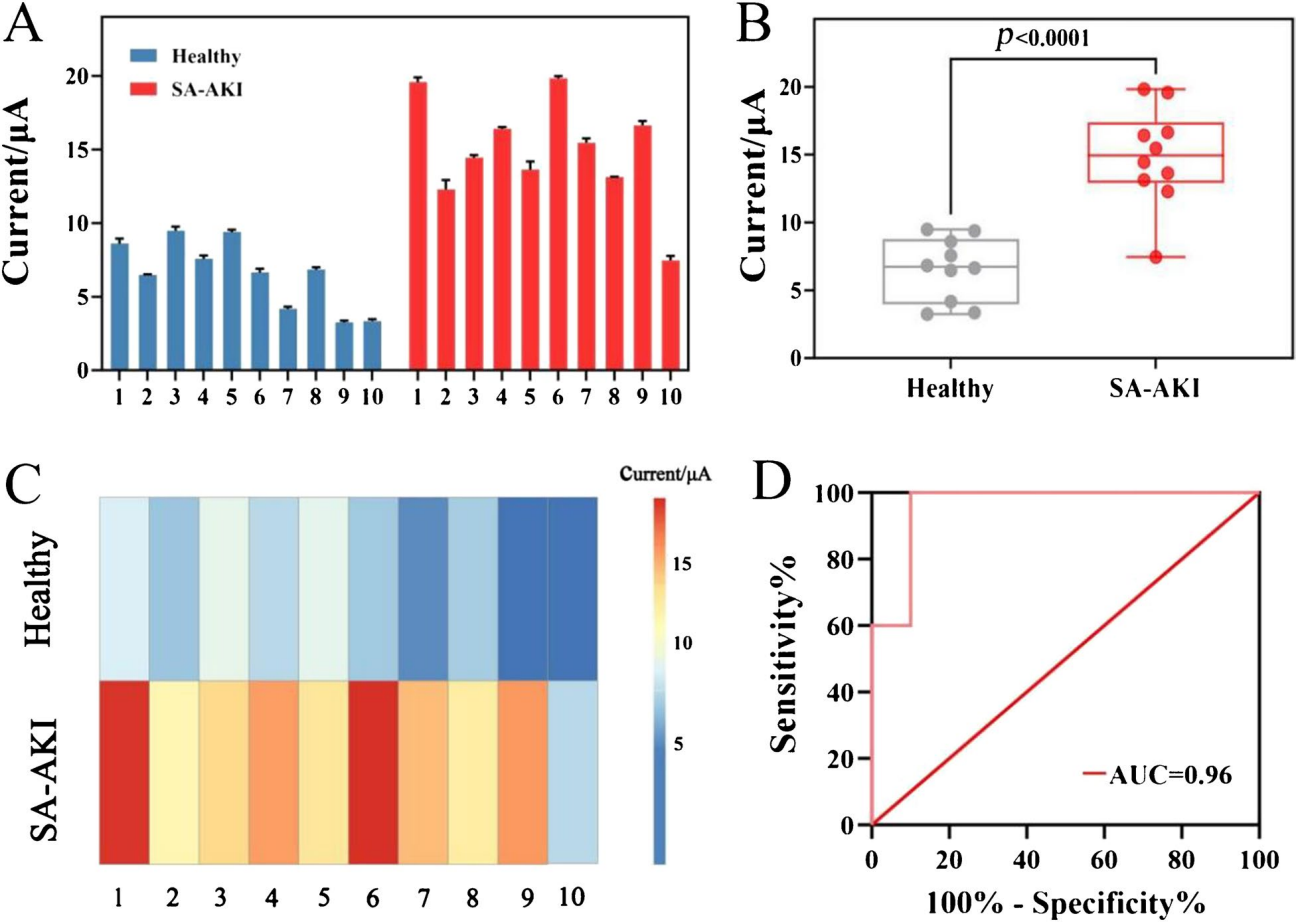
(**A**) Current responses of healthy and SA-AKI groups. (**B**) Boxplot and (**C**) heat map of difference in current response between SA-AKI group and healthy group. (**D**) ROC curve of the electrochemical biosensing method for the diagnosis of SA-AKI

Additionally, clinical serum samples were collected from patients clinically diagnosed with SA-AKI as well as from healthy individuals, and subsequently analyzed using the developed biosensor. Figure 6A displayed the electrochemical signals of the biosensor obtained from the analysis of collected serum samples. It was evident that the electrochemical signals response to the serum samples of SA-AKI group were uniformly higher than those of healthy group. To visualize the distribution of current responses across healthy and SA-AKI groups, a boxplot was presented in Fig. 6B. The median current value was notably higher in SA-AKI group compared to healthy group, with significant disparities (*P* < 0.0001) observed between the two groups. As shown in Fig. 6C, the heat map was further employed to delineate the pronounced dichotomy between healthy group (up panel) and SA-AKI group (down panel). SA-AKI group samples exhibited intensified red hues, whereas healthy group samples consistently displayed blue-dominated profiles. Moreover, a receiver operating characteristic (ROC) curve was constructed to evaluate the practicality of biosensor in SA-AKI diagnosis (n=20, 10 Healthy donors and 10 SA-AKI donors). The area under the ROC curve (AUC) was calculated as 0.96, indicating high diagnostic accuracy.

## Conclusions

In summary, this work successfully developed a highly sensitive electrochemical biosensor for the detection of serum miR-452-5p, a potential biomarker of SA-AKI, based on the Ti_3_C_2_T_x_ MXene-mediated cascading catalytic amplification and CHA-mediated isothermal amplification amplification. The Ti_3_C_2_T_x_ MXene nanozymes possessed excellent peroxidase-like catalytic activity with a *V*_max_ of 9.108×10^−8^ M/s and a *K*_m_ of 22.547 mM for H_2_O_2_, which were slightly inferior to those of natural horseradish peroxidase. The electrochemical biosensor demonstrated high sensitivity for miR-452-5p detection with a LOD as low as 0.09 fM and exhibited good high specificity in a complex matrix. Importantly, this biosensing method achieved a diagnostic accuracy of 96% in clinical samples for direct serum miR-452-5p detection, which held great potential and significance for the early and accurate diagnosis of SA-AKI. It thereby offered promising prospects for related medical research and clinical applications, as well as laid a foundation for the development of portable diagnostic devices. However, the clinical samples in this study were limited, and the long-term stability of this method remained to be further verified.

Clinical Trial Number

Clinical serum samples were obtained after approval by the Institutional Review Board of the University-Town Hospital of Chongqing Medical University (LL-202335).

## Supplementary Information

Below is the link to the electronic supplementary material.

Supplementary file1 (DOCX 326 KB)

## Data Availability

No datasets were generated or analyzed during the current study.
