# Supplementary material for: 2D Ti3C2Tx MXene nanozymes based electrochemical MicroRNA biosensor and application to early diagnosis of sepsis-associated acute kidney injury
Source: Mikrochim Acta. 2025 Nov 1;192(11):777. doi: 10.1007/s00604-025-07559-2 (PMC12579669; doi:10.1007/s00604-025-07559-2)
Supplement: Supplementary file 1 — Supplementary file1 (DOCX 326 KB) [file 604_2025_7559_MOESM1_ESM.docx]

**Supporting Information for**

**2D Ti_3_C_2_T_x_ MXene Nanozymes Based** **Electrochemical MicroRNA Biosensor and Application to Early Diagnosis of Sepsis-Associated Acute Kidney Injury**

Xiangyu Deng^1#^, Xia Zheng^1#^, Anyi Chen^2#^, Yuqing Li^3^, Siling Chen^1^, Jiangling Wu^1^, Jianjiang Xue^1^, Rongjun Yu^1*^, Min Zhao^3^[[1]](#footnote-0)^*^, Jingfu Qiu^2*^

*^1^ Department of Clinical Laboratory, University-Town Hospital of Chongqing Medical University, Chongqing, 401331, China.*

*^2^ College of Public Health, Chongqing Medical University, Chongqing, 400016, China.*

*^3^ Key Laboratory of Clinical Laboratory Diagnostics (Ministry of Education), College of Laboratory Medicine, Chongqing Medical University, Chongqing, 400016, China.*

**Table of contents**

**S1. Reagents and materials**

**S2. Buffers**

**S3. Apparatus**

**S4. Synthesis of AuNPs**

**S5. Electrochemical measurement**

**Fig. S1** Energy dispersive X-ray spectroscopy (EDS) patterns and elemental composition of Ti_3_C_2_T_x_ MXene.

**Fig. S2** SEM images of (A) Ti_3_C_2_T_x_ MXene and (B) AuNPs/Ti_3_C_2_T_x_ MXene.

**Fig. S3** Optimization of the reaction temperature of Ti_3_C_2_T_x_ MXene-TMB-H_2_O_2_ ternary system.

**Fig. S4** Steady-state kinetics assay of Ti_3_C_2_T_x_ Mxene in various concentrations of TMB.

**Fig. S5** PAGE characterization of CHA amplification strategy.

**Fig. S6** Optimization of experimental conditions for the biosensing detection of miR-452-5p.

**Table S1** Sequences of oligonucleotides used in this work.

**Table S2** Comparison of kinetic parameters of Ti_3_C_2_T_x_ Mxene with HRP and other nanozymes.

**Table S3** Comparison of the developed biosensor with other electrochemical methods for miRNAs detection.

**S1. Reagents and materials**

Ti_3_C_2_T_x_ MXene dispersion solution (Lateral size 2~5 µm) was purchased from Jiangsu XFNANO Materials Tech. Co., Ltd. (Nanjing, China). Nafion, phosphorylcholine chloride and choline were obtained from Aladdin Biochemical Tech. Co., Ltd. (Shanghai, China). Gold chloride (HAuCl_4_∙4H_2_O), sodium citrate, 6-mercaptohexanol (MCH), diethanolamine (DEA), hydrogen peroxide (H_2_O_2_, 30%) and 3,3’,5,5’-tetramethylbenzidine (TMB) were purchased from Sigma-Aldrich Chemical (St. Louis, USA). Streptavidin-alkaline phosphatase (SA-ALP) and diethyl pyrocarbonate-treated deionized (DEPC) water were obtained from Sangon Biotech. Co., Ltd. (Chongqing, China). Clinical serum samples were provided by the University-Town Hospital of Chongqing Medical University (Chongqing, China). Meanwhile this project was under the approval of the institutional review board at the Ethics Committee of University-Town Hospital of Chongqing Medical University. To minimize the effect of RNases on the stability of miRNAs, DEPC water was used for all samples and buffer preparations. All the consumables and equipment involved were treated with RNase removal. All synthetic DNA and RNA oligonucleotides were ordered from Sangon Biotech. Co., Ltd. (Chongqing, China) and the sequences were listed in Table S1.

**S2. Buffers**

The buffers involved in this study were as follows: DNA and RNA preparation solution (20 mM Tris-HCl (RNase free) containing 5 mM KCl, 140 mM NaCl, 1 mM MgCl_2_ and 1 mM CaCl_2_, pH 7.4). Washing buffer (Tris buffer including 20 mM Tris-HCl (RNase free), 100 mM NaCl, 5 mM MgCl_2_, pH 7.4). DEA buffer (0.1 M diethanolamine, 100 mM KCl and 1 mM MgCl_2_, pH 9.6). Deionized water (18.2 MΩ/cm) obtained from a Millipore Mill-Q purification system was used for all solution preparation.

**S3. Apparatus**

Electrochemical measurements, including DPV, CV and EIS were all implemented on an AUTOLAB PGSTAT302 N electrochemical workstation (Metrohm Technology Co. Ltd., Switzerland). The electrochemical workstation operated based on a conventional three-electrode system comprising of a saturated calomel electrode (as the reference electrode), a platinum wire (as the auxiliary electrode) and a 3-mm diameter glassy carbon electrode (as the working electrode). A JY600C electrophoresis analyzer (Beijing Junyi Electrophoresis Co. Ltd., China) was used to perform gel electrophoresis experiments, and gel imaging was carried out on a G: BOX F3 GENE Sys (Gene Co. Ltd., China). UV-Vis absorbance spectra were carried out by a UV-1750 UV-Vis spectrophotometer (SHIMADZU Co., Ltd., Japan). The transmission electron microscope (TEM) image were acquired using TEM (FEI Tecnai G2 F30, NL). Energy dispersive X-ray spectroscopy (EDS) was performed with a JEOL JSM-6700F microscope (Japan).

**S4. Synthesis of AuNPs**

Briefly, the sodium citrate solution (2.0 mL, 1%) was rapidly added to the boiling HAuCl_4_ solution (100 mL, 0.01%) under magnetic stirring and maintained at 100 °C for 15 min. After the color changed from gray to wine-red, the mixture was cooled to room temperature with continuous stirring. The products were stored at 4°C in darkness for further use.

**S5.** **Electrochemical measurement**

The CV measurements were conducted by scanning the voltage range from -0.4 V to 0.8 V at a scan rate of 0.1 V/s. An amplitude of 0.01 V and a frequency sweep range of 10^-1^ Hz to 10^5^ Hz, such parameters were used for EIS measurements. The CV and EIS measurements were implemented in 5 mM [Fe(CN)_6_]^3-/4-^ containing 0.1 M KCl. The DPV measurements were performed using voltages ranging from -0.15 to 0.3 V, modulation amplitudes of 0.07 V, modulation time of 0.05 s and interval time of 0.2 s. All measurements were carried out at room temperature.


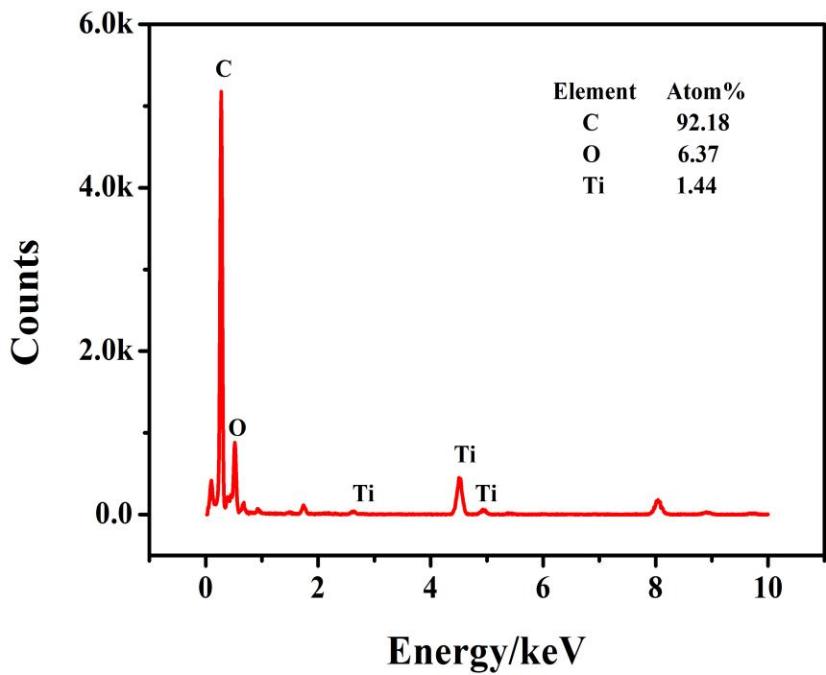


**Fig. S1** Energy dispersive X-ray spectroscopy (EDS) patterns and elemental composition of Ti_3_C_2_T_x_ MXene.


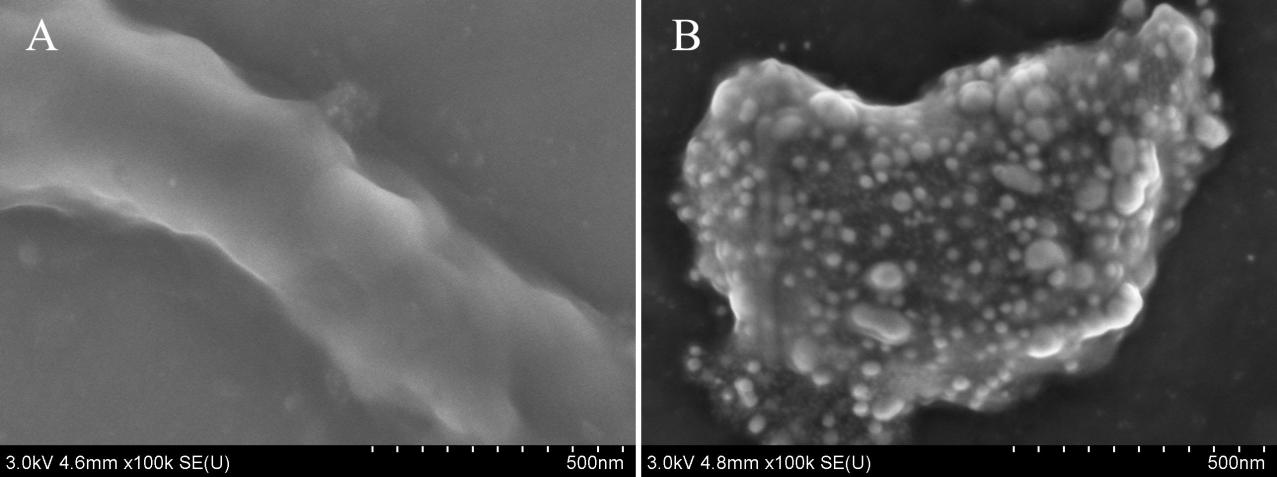


**Fig. S2.** SEM images of (A) Ti_3_C_2_T_x_ MXene and (B) AuNPs/Ti_3_C_2_T_x_ MXene.


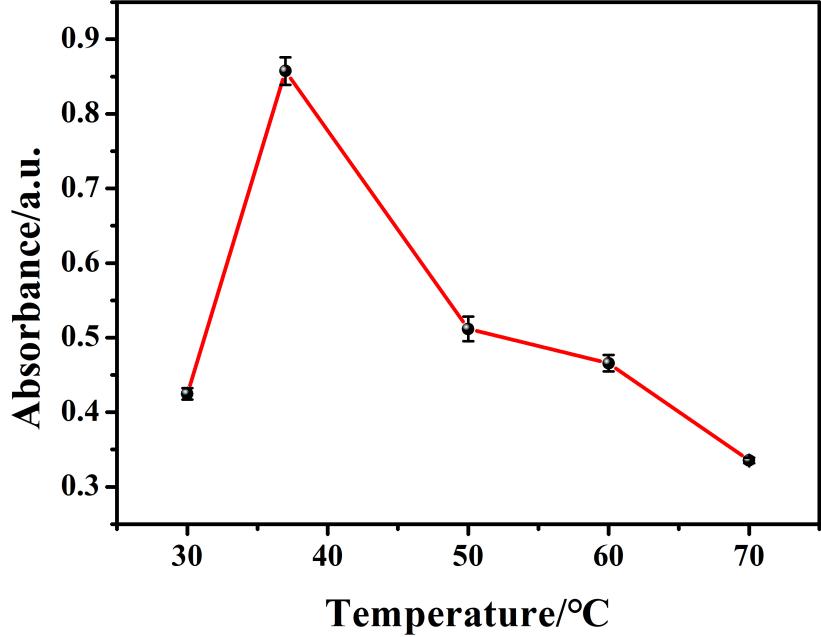


**Fig. S3.** UV–Vis absorption peak at 652 nm of Ti_3_C_2_T_x_ MXene-TMB-H_2_O_2_ ternary system in pH 3.6 at different temperatures.


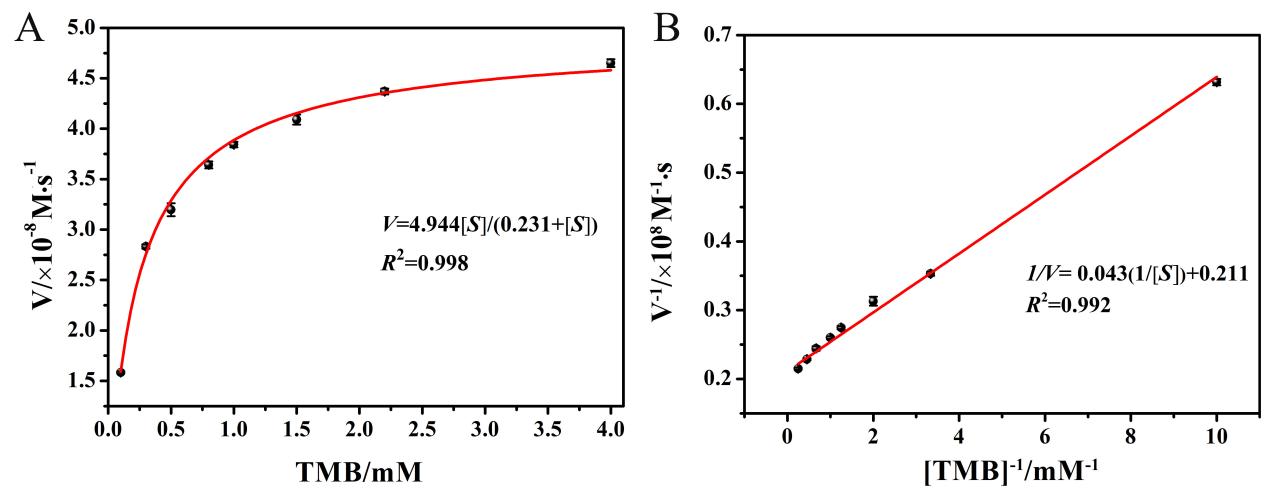


**Fig. S4** (A) Steady-state kinetics assay and (D) corresponding double-reciprocal plots of Ti_3_C_2_T_x_ MXene (33.0 µg/mL) with different concentrations of TMB (0.1, 0.3, 0.5, 0.8, 1.0, 1.5, 2.2, 4.0 mM).

**PAGE characterization of CHA amplification strategy**

To validate the successful assembly of CHA, a 20% native PAGE was conducted to substantiate the interactions between hairpin DNAs and miR-452-5p. As depicted in Fig. S5, it could be seen that the presence of bands in lane 1, lane 2 and lane 3, which were correspond to the miR-452-5p (target), H1 and H2, respectively. Subsequently, a new band corresponding to the H1-miR-452-5p complex emerged at a reduced electrophoresis distance, while the bands associated with H1 and miR-452-5p were almost completely diminished (Lane 4), indicating successful opening of the hairpin structure of H1 and subsequent binding. In absence of miR-452-5p, no significant formation of H1-H2 complex was observed when incubating H1 with H2 at 37 ℃ for 2 h (lane 5), suggesting absence of hybridization between these two hairpin DNAs. However, upon addition of target miR-452-5p to the mixture of H1 and H2, a new band corresponding to the H1-H2 complexes appeared closer to the anode, whereas the bands related to both H1 and H2 were nearly entirely vanished (lane 6), thereby confirming efficient miR-452-5p-mediated catalytic hairpin assembly.


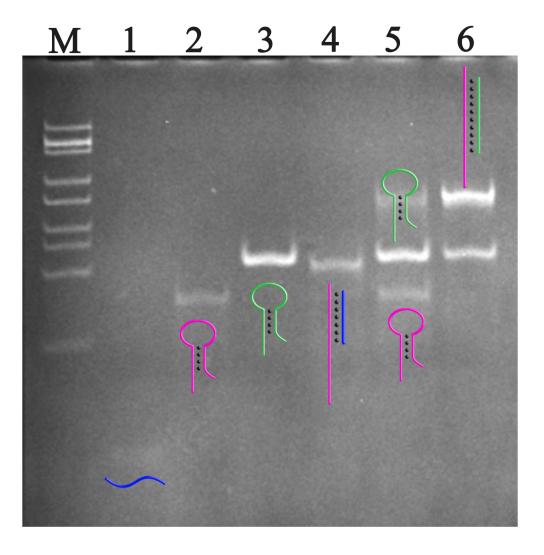


**Fig. S5** Native PAGE image of different samples: (1) Target, (2) H1, (3) H2, (4) H1+target, (5) H1+H2, (6) H1+H2+target. The concentrations of hairpin DNAs and target miR-452-5p strands were 2.0 µM.


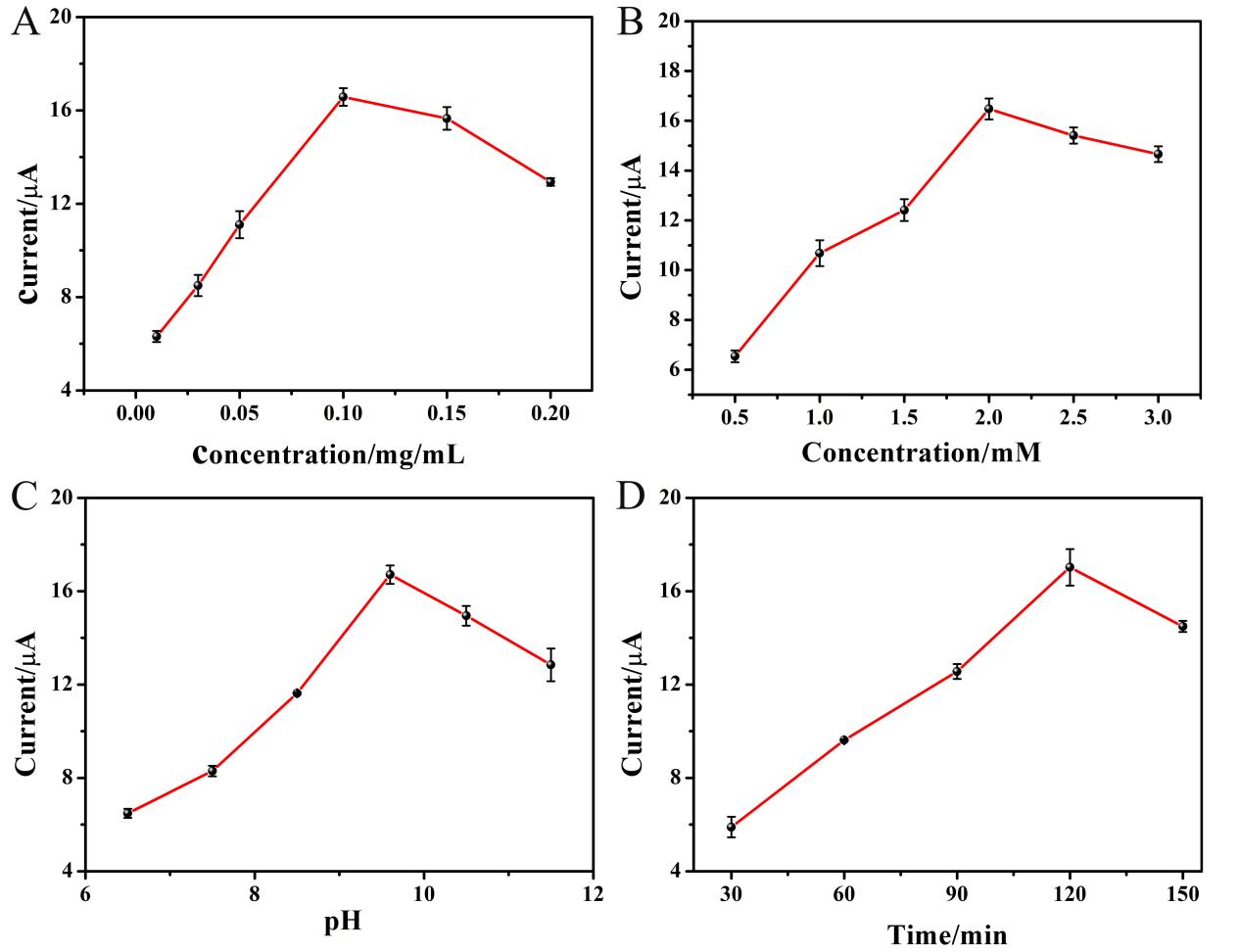


**Fig. S6** Optimization of experimental conditions: (A) Ti_3_C_2_T_x_ MXene concentration, (B) H1 concentration, (C) pH of DEA buffer, (D) CHA reaction time. The error bars represent the standard deviation of three parallel measurements.

**Table S1.** Sequences of oligonucleotides used in this work.

| **Oligonucleotides** | **Sequence (from 5' to 3')** |
| --- | --- |
| miR-452-5p | AAC UGU UUG CAG AGG AAA CUG A |
| H1 | SH-TTT TTT TCA GTT TCC TCT GCA AAC AGT TTA CCG TGC TTG CAA CTG TTT GCA GAG G |
| H2 | GTT ATT AAT GTG TGA TGT TGC AAG CAC GGT AAA CTG TTT GCA GAG GAA TCA GAG AAA CAG TTT ACC GTG |
| SP | ACA TCA CAC ATT A AT A AC CTA CCA TCG CAC TTA GAT-Biotin |
| M1 | AAC UGU UUG CUG AGG AAA CUG A |
| M2 | AAC UGU UUG CUG AGG UAA CUG A |
| miR-21 | UAG CUU AUC AGA CUG AUG UUG A |
| miR-146 | UGA GAA CUG AAU UCC AUG GGU U |

M1 and M2 were single-base mismatched miRNA, double-base mismatched miRNA, respectively.

**Table S2** Comparison of kinetic parameters of Ti_3_C_2_T_x_ Mxene with HRP and other nanozymes.

| **Catalyst** | ***K*_m_/mM** | | ***V*_max_/10^-8^ M/s** | | **Ref.** |
| --- | --- | --- | --- | --- | --- |
|  | **H_2_O_2_** | **TMB** | **H_2_O_2_** | **TMB** |  |
| HPR | 3.7 | 0.434 | 8.71 | 10.0 | [1] |
| Fe_3_O_4_ MNPs | 154 | 0.098 | 9.78 | 3.44 | [1] |
| C-Dots | 26.77 | 0.039 | 30.61 | 3.61 | [2] |
| PtNTs | 155 | 0.0186 | 24.66 | 11.79 | [3] |
| **Ti_3_C_2_T_x_ Mxene** | **22.547** | **0.231** | **9.108** | **4.944** | **This work** |

**Table S3** Comparison of the developed biosensor with other electrochemical methods for miRNAs detection.

| **Amplification strategy** | **Linear range (fM)** | **LOD (fM)** | **Ref.** |
| --- | --- | --- | --- |
| Fe_3_O_4_/CeO_2_@Au | 1.0~1.0×10^6^ | 0.33 | [4] |
| Carbon black and Au NPs | 2.9~7.0×10^8^ | 1.0 | [5] |
| N-carboxymethyl chitosan/molybdenum carbide | 1.0~1.0×10^6^ | 0.34 | [6] |
| Au@MoS_2_ nanosheet | 10~1.0×10^6^ | 0.45 | [7] |
| **Ti_3_C_2_T_x_ MXene nanozyme**  **and CHA** | **0.1~1.0×10^7^** | **0.09** | **This work** |

**References**

(1) Gao, L.; Zhuang, J.; Nie, L.; Zhang, J.; Zhang, Y.; Gu, N.; Wang, T.; Feng, J.; Yang, D.; Perrett, S.; Yan, X. Intrinsic Peroxidase-like Activity of Ferromagnetic Nanoparticles. *Nature Nanotechnology* **2007**, *2* (9), 577–583.

(2) Shi, W. B.; Wang, Q. L.; Long, Y. J.; Cheng, Z. L.; Chen, S. H.; Zheng, H. Z.; Huang, Y. M. Carbon nanodots as peroxidase mimetics and their applications to glucose detection. *Chemical Communications* **2011**, 47, 6695–6697.

(3) Cai, K.; Lv, Z. C.; Chen, K.; Huang, L.; Wang, J.; Shao, F.; Wang, Y. J.; Han, H. Y. Aqueous synthesis of porous platinum nanotubes at room temperature and their intrinsic peroxidase-like activity. *Chemical Communications* **2013**, 49,6024–6026.

(4) Liu, S.; Yang, Z.; Chang, Y.; Chai, Y.; Yuan, R. An Enzyme-Free Electrochemical Biosensor Combining Target Recycling with Fe_3_O_4_/CeO_2_@Au Nanocatalysts for microRNA-21 Detection. *Biosensors and Bioelectronics* **2018**, *119*, 170–175.

(5) Yammouri, G.; Mohammadi, H.; Amine, A. A Highly Sensitive Electrochemical Biosensor Based on Carbon Black and Gold Nanoparticles Modified Pencil Graphite Electrode for microRNA-21 Detection. *Chemistry Africa* **2019**, *2* (2), 291–300.

(6) Tian, L.; Qi, J.; Ma, X.; Wang, X.; Yao, C.; Song, W.; Wang, Y. A Facile DNA Strand Displacement Reaction Sensing Strategy of Electrochemical Biosensor Based on N-Carboxymethyl Chitosan/Molybdenum Carbide Nanocomposite for microRNA-21 Detection. *Biosensors and Bioelectronics* **2018**, *122*, 43–50.

(7) Su, S.; Cao, W.; Liu, W.; Lu, Z.; Zhu, D.; Chao, J.; Weng, L.; Wang, L.; Fan, C.; Wang, L. Dual-Mode Electrochemical Analysis of microRNA-21 Using Gold Nanoparticle-Decorated MoS_2_ Nanosheet. *Biosensors and Bioelectronics* **2017**, *94*, 552–559.

1. *Corresponding author

   E-mail address: [yurongjun@hospital.cqmu.edu.cn](mailto:yurongjun@hospital.cqmu.edu.cn) (R. Yu), [zhaomin@cqmu.edu.cn](mailto:chenay@cqmu.edu.cn) (M. Zhao), jfqiu@126.com (J. Qiu).

   ^#^ These authors contributed equally to this work. [↑](#footnote-ref-0)
